# Dense Paraphrasing for multimodal dialogue interpretation

**DOI:** 10.3389/frai.2024.1479905

**Published:** 2024-12-19

**Authors:** Jingxuan Tu, Kyeongmin Rim, Bingyang Ye, Kenneth Lai, James Pustejovsky

**Affiliations:** Computer Science Department, Brandeis University, Waltham, MA, United States

**Keywords:** Dense Paraphrasing, Common Ground Tracking, dialogue system, Large Language Models, multimodal communication

## Abstract

Multimodal dialogue involving multiple participants presents complex computational challenges, primarily due to the rich interplay of diverse communicative modalities including speech, gesture, action, and gaze. These modalities interact in complex ways that traditional dialogue systems often struggle to accurately track and interpret. To address these challenges, we extend the textual enrichment strategy of Dense Paraphrasing (DP), by translating each nonverbal modality into linguistic expressions. By normalizing multimodal information into a language-based form, we hope to both simplify the representation for and enhance the computational understanding of situated dialogues. We show the effectiveness of the dense paraphrased language form by evaluating instruction-tuned Large Language Models (LLMs) against the Common Ground Tracking (CGT) problem using a publicly available collaborative problem-solving dialogue dataset. Instead of using multimodal LLMs, the dense paraphrasing technique represents the dialogue information from multiple modalities in a compact and structured machine-readable text format that can be directly processed by the language-only models. We leverage the capability of LLMs to transform machine-readable paraphrases into human-readable paraphrases, and show that this process can further improve the result on the CGT task. Overall, the results show that augmenting the context with dense paraphrasing effectively facilitates the LLMs' alignment of information from multiple modalities, and in turn largely improves the performance of common ground reasoning over the baselines. Our proposed pipeline with original utterances as input context already achieves comparable results to the baseline that utilized decontextualized utterances which contain rich coreference information. When also using the decontextualized input, our pipeline largely improves the performance of common ground reasoning over the baselines. We discuss the potential of DP to create a robust model that can effectively interpret and integrate the subtleties of multimodal communication, thereby improving dialogue system performance in real-world settings.

## 1 Introduction

Modeling the interpretation of multimodal dialogue remains a challenging task, both formally and computationally (Saha et al., 2018; Liao et al., 2018). It involves not only aligning and composing the meanings conveyed through the different modalities, such as speech, gesture, and gaze, but also identifying actions and contextual factors occuring during the interaction. Traditionally, dialogue systems have had difficulty tracking and interpreting the diverse interactions between multiple communicative modalities, particularly when faced with the problem of underspecified references (Vinyals and Le, 2015; Baltrušaitis et al., 2018).

When engaged in dialogue, our shared understanding of both utterance meaning (content) and the speaker's meaning in a specific context (intent) involves the ability to link these two in the act of situationally grounding meaning to the local context—what is typically referred to as “establishing the common ground” between speakers (Clark and Brennan, 1991; Traum, 1994; Asher and Gillies, 2003; Dillenbourg and Traum, 2006). The concept of common ground refers to the set of shared beliefs among participants in Human-Human interaction (HHI) (Traum, 1994; Hadley et al., 2022), as well as Human-Computer Interaction (HCI) (Krishnaswamy and Pustejovsky, 2019; Ohmer et al., 2022) and Human-Robot Interaction (HRI) (Kruijff et al., 2010; Fischer, 2011; Scheutz et al., 2011). Researchers have recently employed the notion of common ground operationally to identify and select relevant information for conversational Question Answering (QA) system design (Nishida, 2018; Del Tredici et al., 2022).

In conversational multimodal dialogue systems, it is not enough to simply recognize individual modalities, such as speech, gesture, or gaze, in isolation. The true challenge lies in the accurate alignment and integration of these modalities to derive a cohesive understanding of the dialogue context. For instance, the subtle yet critical co-attention between participants—where both parties focus on the same object or region of interest—can dramatically shift the meaning of an utterance. If a system fails to detect or properly integrate these multimodal cues, the resulting interpretation may be incomplete or even incorrect, leading to misunderstandings and breakdowns in communication.

Underspecified references, such as pronouns and demonstratives, are frequently used in natural conversation to refer to entities that are contextually salient but not explicitly named. This reliance on shared context can lead to ambiguities that are challenging for dialogue systems to resolve (Byron, 2002; Eckert and Strube, 2000; Müller, 2008; Khosla et al., 2021).

**Figure 1:**
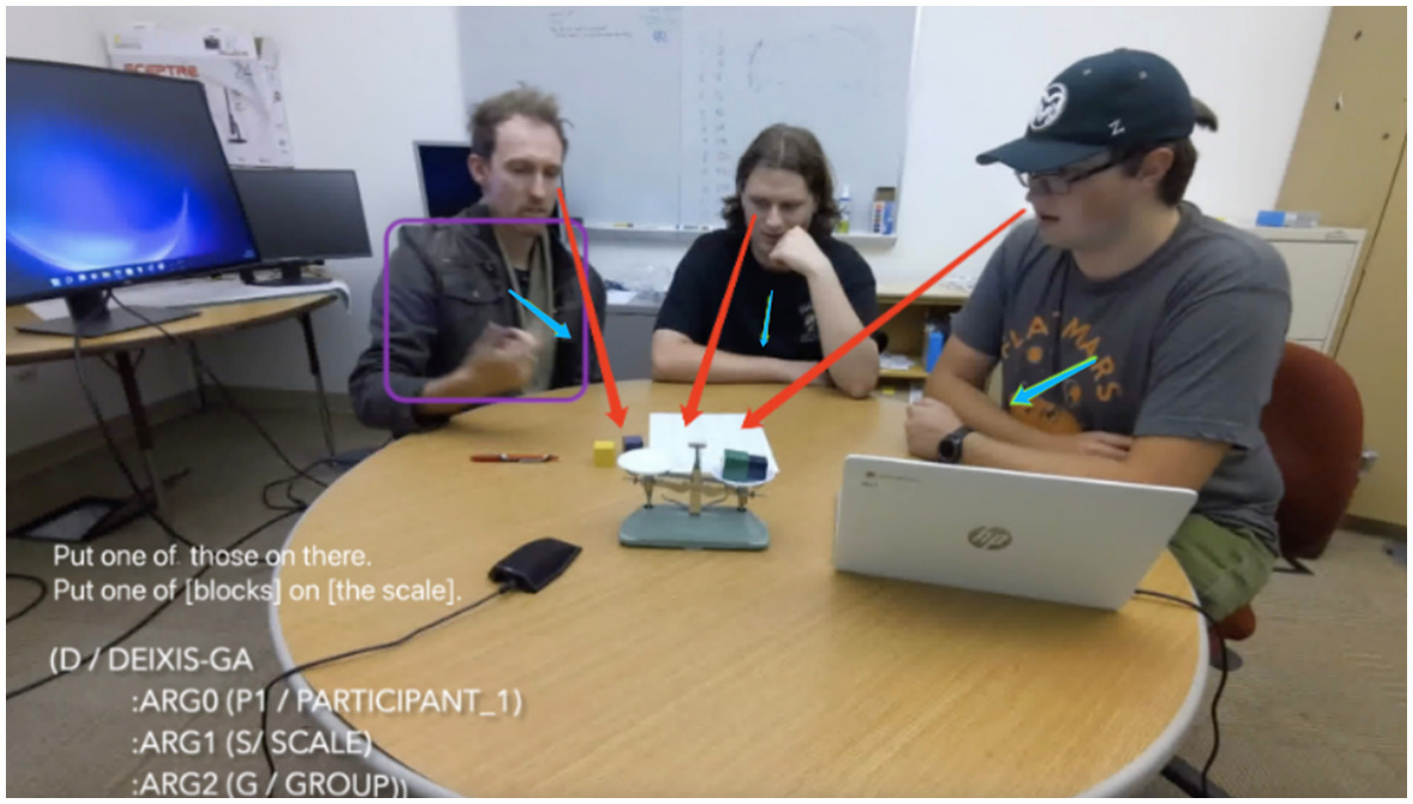
Example of a triad (three participants, P1, P2, P3) multimodal interaction in the weights task: P1 (left) says: “Put one of those on there.”; purple box denotes P1 pointing to the blocks and scale; red arrows denote co-gazing by P1–P3; blue arrows symbolize P1–P3 leaning toward the table.

For example, when a speaker says “one of *those*” while pointing at an object, as in Figure 1, the word itself is insufficient to convey the full meaning without considering the accompanying gesture. The integration of visual cues from gestures and gaze with linguistic information allows the system to disambiguate these references by narrowing down the possible entities being referred to. Moreover, the synchronization of gestures with speech provides additional semantic information, such as emphasis or referential clarification (e.g., the locational demonstrative *there* in Figure 1), that is crucial for understanding the speaker's intent.

Consequently, the need for more robust methods to handle these ambiguities is of great importance. Advanced Artificial Intelligence (AI) systems must incorporate sophisticated multimodal fusion techniques that not only recognize each modality but also align and integrate them to form a unified representation of the dialogue context. This process involves leveraging models that can map gestures to referential expressions, correlate gaze patterns with attentional focus, and link these nonverbal cues with the linguistic content of the conversation.

To address this challenge, our research adopts the data augmentation technique of Dense Paraphrasing (DP) (Tu et al., 2023; Rim et al., 2023) to the task of interpreting multimodal dialogue. In this extension, we propose Multi-Modal Dense Paraphrasing (MMDP) that involves translating nonverbal modalities into linguistic expressions, thereby recontextualizing and clarifying the meaning of underspecified references. By creating cross-modal coreference links and binding these references with action or gesture annotations, we aim to enrich the textual content and enhance the computational understanding of dialogues.

We explore the utility of MMDP on the Common Ground Tracking (CGT) problem (Khebour et al., 2024) on the recent published Weights Task Dataset (WTD) (Khebour et al., 2023). This dataset contains videos in which groups of three were asked to determine the weights of five blocks using a balance scale. This collection contains annotations from multiple modalities recorded in the videos, as well as identification of the group epistemic state at each dialogue state. The CGT problem defined over the dataset is to identify the common ground (knowledge of the weights of different blocks) among the participants of each group. In our previous joint work (Khebour et al., 2024), a hybrid method of neural networks and heuristics was adopted to solve the CGT problem.

In this paper, we instead treat CGT as a QA task that involves two steps: applying MMDP to convert information from multiple modalities into meaningful paraphrases, and then using the paraphrases as the context for prompts that ask about the common ground. We leverage Large Language Models (LLMs) for the whole pipeline and evaluate the results under different settings. We find that the human readable paraphrase generated by MMDP can better integrate the information from the dialogue context and multiple modalities, thus improving the performance over baselines by a large margin. We also compare the results by varying different models and the length of input context, providing further insights for future work. We make our source code and data publicly available.^1^

## 2 Related work

Recent years have seen remarkable progress on tasks involving multimodality (Chhabra and Vishwakarma, 2023; Das and Singh, 2023; Zhao et al., 2023; Gong et al., 2023). Encoding multimodal information into embeddings involves combining data from different modalities, such as text, images, and audio, into a unified representation, and is a vital component of many multimodal tasks.

In recent studies, multimodal encoders are usually built upon different vector extraction algorithms for different modalities, and then a combination operation is performed over those vectors. For example, to combine language and vision modalities, Chuang et al. (2020) use contextualized word embeddings for language and acoustic feature extraction for audio, and then uses vector addition of the two to train an RNN model. Similarly, Surís et al. (2018) leverage two separate video and audio features to train shared weights. On the other hand, Khebour et al. (2024) use concatenation of word embeddings and more transparent k-hot encodings to encode multimodal information. More recently, multimodal LLMs such as GPT-4V (OpenAI et al., 2024) have been used to incorporate image inputs into LLMs. Contrary to previous studies, in this paper, we leverage LLMs to map non-verbal modality data into a natural language form and then treat the text as augmented multimodal data.

QA is a significant area in NLP and various other NLP tasks such as Summarization (Eyal et al., 2019; Deutsch et al., 2021; Gunasekara et al., 2021), Data Augmentation (Mekala et al., 2022), and Question Generation (Tu et al., 2022b) can be enhanced by integrating QA techniques. We leverage QA to facilitate the tracking of common ground in situated dialogue in this work. The general goal of Dialogue State Tracking (DST) is to maintain and update the state of dialogue by accurately tracking user intents and belief states during a multi-turn conversation (Budzianowski et al., 2018; Liao et al., 2021; Jacqmin et al., 2022). Del Tredici et al. (2022) introduce CGT as a mitigation method for conversational QA. The task aims to estimate the shared understanding or “common ground” between the conversational participants. DST focuses on task completion within a single session and deals with specific slots and intents related to the task, while CGT focuses on maintaining mutual understanding throughout the conversation with broader shared knowledge and assumptions. Khebour et al. (2024) is the first attempt to apply CGT over real-world multiparty dialogue instead of just conversational QA.

Textual enrichment has been employed to address the challenge of understanding the economy of sentence structure in comprehension tasks. Approaches to textual enrichment include paraphrasing (Bhagat and Hovy, 2013; Barzilay and Elhadad, 1997) and decontextualization (Choi et al., 2021; Elazar et al., 2021; Wu et al., 2021). DP has been recently introduced in Tu et al. (2023) as a linguistically motivated textual enrichment strategy and has been leveraged to facilitate a variety of NLP tasks such as Coreference Resolution (Rim et al., 2023), Completion (Ye B. et al., 2022), and Meaning Representation (Tu et al., 2024). Khebour et al. (2024) also used DP to recover propositional content (and subsequent sentence embeddings) in user utterances in multimodal data. We further extend the usage of DP to translate nonverbal modalities into linguistic expressions in the broader context of Natural Language Generation (NLG), the task of generating natural language text from a knowledge base or logical form representation. NLG is a crucial component of QA and dialogue systems. Traditional NLG methods are mostly rule-based (Bateman and Henschel, 1999; Busemann and Horacek, 1998), while later works approach the problem with neural networks (Zhou et al., 2016; Tran and Nguyen, 2018). With the recent advances in LLMs, such models (Touvron et al., 2023; Achiam et al., 2023) show great capabilities in generation tasks. In this paper, we leverage LLMs to facilitate DP and generate answers for CGT questions.

## 3 Theory and practice of dense paraphrasing

In this section, we introduce the textual enrichment and data augmentation strategy of Dense Paraphrasing (DP), and describe how it enables deeper capabilities in computational Natural Language Understanding (NLU) models.

### 3.1 Background and definition

NLU has long been considered a fundamental task within AI, involving both parsing and understanding the semantics of language inputs, including grammar, context, and intent. Such work has focused on enabling machines to perform tasks like sentiment analysis, question answering, information extraction, and information retrieval effectively.

NLU, however, remains an extremely difficult task, particularly when deployed in the service of dialogue understanding and conversation analysis (Ye F. et al., 2022; Yi et al., 2024; Ou et al., 2024).

Furthermore, despite the fast-paced growth of AI, advanced computational models are still challenged by natural language partly due to lacking a deeper understanding of the economy of sentence structures. We, as humans, interpret sentences as contextualized components of a narrative or discourse, by both filling in missing information, and reasoning about event consequences. However, most existing language models understand inferences from text merely by recovering surface arguments, adjuncts, or strings associated with the query terms or prompts (Parikh et al., 2016; Chen et al., 2017; Kumar and Talukdar, 2020; Schick and Schütze, 2021).

Prior work on improving NLU systems to learn beyond the surface texts has taken two directions. The first involves commonsense reasoning and knowledge understanding (Poria et al., 2014; Angeli and Manning, 2014; Emami et al., 2018; Mao et al., 2019; Lin et al., 2021), both of which improve NLU models by providing the ability to make inferences and interpret nuances from knowledge about the everyday world, and concepts of entities from knowledge bases.

The second line of work involves data augmentation over the input. This approach focuses on paraphrasing or enriching the texts by increasing the variability in the text format, and reducing the dependency on the contexts from other texts (Culicover, 1968; Goldman, 1977; Muraki, 1982; Boyer and Lapalme, 1985; McKeown, 1983; Barzilay and Elhadad, 1997; Bhagat and Hovy, 2013; Choi et al., 2021; Elazar et al., 2021; Chai et al., 2022; Eisenstein et al., 2022; Tu et al., 2022b; Ye B. et al., 2022; Katz et al., 2022). We argue here that such augmented texts can in turn help NLU systems to better handle the ambiguities and variants in human language, particularly when used in multimodal settings. We extend the technique of Dense Paraphrasing (DP) (Tu et al., 2023) to multimodal interactions. DP is a technique that rewrites a textual expression to reduce ambiguity while making explicit the underlying semantics of the expression. DP reveals a set of paraphrases that act as the signature for a semantic type, which is consistent with canonical syntactic forms for a semantic type (Pustejovsky, 1995). Here we define DP as follows:

** Definition 1**. **Dense Paraphrasing** **(DP)**: Given a pair (*S, P*) of two expressions in a language, *P* is a valid *Dense Paraphrase* of *S* if *P* is an expression (lexeme, phrase, sentence) that, (1) **[consistency]** eliminates any contextual ambiguity that may be present in *S*; (2) **[informativeness]** makes explicit any underlying semantics (hidden arguments, dropped objects or adjuncts) that is not otherwise expressed in the economy of sentence structure.

### 3.2 Subtasks of Dense Paraphrasing

In practice, to achieve the said level of context-independence and generate fully self-sustained textual expressions, we include (but are not limited to) the following subtasks as the fundamental building blocks of DP augmentation:

**Anaphora and coreference**: Understanding the contextual semantics of referring expressions is a crucial step for NLU. To that end, being able to dereference and then to canonicalize pronouns and other noun phrases is an integral step toward DP.

**Frame saturation**: Argument structure in event semantics can provide a rich understanding of relations among event participants and causal relations between entity states (as a result of the event). However, due to the economy of natural language, the full argument structure of an event is seldom present in linguistic surface forms. Hence recovering those omitted arguments and saturating the event frames (argument structures) is another critical goal for DP.

**Event decomposition**: Some events can be decomposed into multiple steps or subevents. Humans can easily understand underlying subevent structures (individual subevents and their temporal order) based on their lexical competence, and hence can use abstract vocabulary for complex actions and events in natural language. Surfacing the underlying subevent structure is another aspect of what DP aims to achieve in terms of data augmentation for NLU systems.

**Entity state tracking**: Actions have consequences. Events make changes to paricipant entities and re-configure the world status. However, for the same economic reason, we humans heavily rely on prior (commonsense or empirical) knowledge to carry complex causal and temporal relations between entities through chains of events. Thus, within DP, we aim to provide temporally ordered state changes as a part of the textual enrichment strategy.

**Multimodal alignment**: Motivated by the concept of DP that is first outlined in and adopted by the above work to create rich paraphrases of implicit entities represented in structured graphs, we extend DP to encode the multimodal input into a *machine readable* format, and then decode it into *human readable* paraphrases. Text in machine readable format is a form of (semi-)structured textual representation of the multimodality that is flexible enough to be ingested by the model and transformed into other formats. Text in human readable format is natural language that is more effectively processed and interpreted by language models. More implementational details are described in Section 6.2.4.

### 3.3 Applications of DP

In previous work, we proposed the textual enrichment strategy called Dense Paraphrasing (DP), and explored how it enables deeper NLU capability for computational models. DP transforms and enriches the texts that will be input to the computational models. It reflects and facilitates the models' capability to understand the meaning of language in a way that improves downstream NLU tasks. DP differs from previous work in that it is more linguistically motivated and focuses on the realization of compositional operations inherent in the meaning of the language. This makes DP-enriched texts independent of external knowledge, relying solely on the contextualized or grounded information from the sentence or document structure.

The proposed DP technique helps address practical NLU tasks by providing tools, datasets, and resources that allow models to learn text more efficiently and easily by augmenting the context with traceable states for all mentions and events involved in the text. Given the context, DP can enrich the text by enriching the events with their implicit state information and linking the enriched events until the goal is reached.

DP has been applied to improve the logical metonymy task by surfacing implicit types through the semantic reconstruction of the sentence (Ye B. et al., 2022). Metonymy identifies implicit meaning, such as the understood activity of “drinking” in *Jon enjoyed his coffee*. The paraphrased sentences with an explicated event-argument structure are used to train masked language models for the logical metonymy task.

Tu et al. (2022a,b) defined a QA task that applies DP to generate questions over implicit arguments and event states from procedural texts, which provided a lens into a model's reasoning capability in the task. The QA task includes competence-based questions that focus on queries over lexical semantic knowledge involving implicit argument and subevent structures of verbs. The paper found that the corresponding QA task is challenging for large pre-trained language models until they are provided with additional contextualized semantic information. Obiso et al. (2024) also demonstrated that QA tasks using DP-enriched contexts leads to increased performance on various models.

The DP technique has been further applied to a more challenging coreference and anaphora resolution task that involves implicit and transformed objects. Tu et al. (2023) applied DP on procedural texts to generate hidden arguments and explicate the transformation of the arguments from a chain of events on the surface texts. Following this, Rim et al. (2023) utilized the proposed event semantics for the entity transformation to represent recipe texts as I/O process graph structures that are able to better model entity coreference.

DP can also be used for constructing novel linguistic resources. Tu et al. (2024) proposed to enrich Abstract Meaning Representation (AMR) with GL-VerbNet. The paper developed a new syntax, concepts, and roles for subevent structure based on VerbNet for connecting subevents to atomic predicates. They demonstrated the application of the new AMR dataset for generating enriched paraphrases with details of subevent transformations and arguments that are not present in the surface form of the texts.

## 4 Common Ground Tracking

Common Ground Tracking (CGT) is the task of identifying the shared belief space held by all participants in a task-oriented dialogue (Khebour et al., 2024). This involves finding the propositions that are acknowledged and accepted by all participants engaged in the task. In this context, we model the dialogue as a set of beliefs and the evidence supporting those beliefs at each conversational turn. Each turn may introduce, reinforce, or change beliefs, and the CGT task focuses on tracking these shared understandings throughout the dialogue. To do this, we use a Common Ground Structure (CGS), inspired by the notion of a dialogue gameboard (Ginzburg, 2012), as well as by evidence-based dynamic epistemic logic (van Benthem et al., 2014; Pacuit, 2017). A CGS has three components (Example usage in Section 5.1):

1. QBank: set of propositions that could be true; i.e., that have not yet been ruled out;
2. EBank: set of propositions for which there is some evidence they are true;
3. FBank: set of propositions believed as true by the group.

To evaluate systems designed for CGT, we formulate it as a QA task. In this setup, the system is prompted with questions that aim to identify the shared beliefs (represented in terms of the contents of the three banks) at each turn in the dialogue along with the current context. By treating CGT as a QA task, we provide a structured method for quantitatively evaluating the effectiveness of systems in tracking and updating shared beliefs among dialogue participants. This formulation not only helps in understanding the common ground reached but also in assessing the implicit and explicit acknowledgment of information as the conversation progresses.

## 5 Dataset

For our experiments, we use the Weights Task Dataset (WTD) (Khebour et al., 2023, 2024). The WTD contains ten videos, totaling ~170 min, in which groups of three were asked to determine the weights of five blocks using a balance scale. During the task, participants communicated with each other using multiple modalities, including language, gesture, gaze, and action. Participants were recruited from a university setting, spoke English, and were between 19 and 35 years of age.

The WTD includes multiple layers of annotations. Speech was segmented and transcribed three ways: automatically, using Google Cloud ASR and Whisper; and manually by humans. Gestures, including deictic (pointing), iconic (depicting properties of objects or actions), and emblematic or conventional gestures, were annotated using Gesture AMR (GAMR) (Brutti et al., 2022; Donatelli et al., 2022). Actions, including participant actions (lifting blocks, or putting them on other objects) and scale actions (whether the scale is balanced, or leaning in some direction), were represented using VoxML (Pustejovsky and Krishnaswamy, 2016). Collaborative problem-solving indicators, measuring ways in which groups share knowledge and skills to jointly solve problems, were annotated using the framework of Sun et al. (2020). The NICE coding scheme (Dey et al., 2023) was used to annotate additional indicators of engagement, including gaze, posture, and emotion. Finally, the WTD contains Common Ground Annotations (CGA); these include dialogue moves, such as STATEMENT (announcement of some proposition), ACCEPT (agreement with a previous statement), and DOUBT (disagreement with a previous statement); and participant observations and inferences that justify statements.

### 5.1 Common ground tracking in the weights task dataset

At the beginning of each Weights Task dialogue, we initialize QBank with propositions, where each proposition states that a certain block (denoted by its color, red, blue, green, purple, or yellow) has a certain weight (between 10 and 50 grams, in 10-gram intervals). With five blocks and five possible weights, QBank contains 5 × 5 = 25 propositions. Meanwhile EBank and FBank are initially empty, as nothing has yet been discussed.

As the dialogue progresses, we update the CGS as follows, according to the CGA. The STATEMENT of a proposition (e.g., *blue is 10*), or of something that would entail it (e.g., *red and blue are equal*, when red = 10 is already in FBank), moves that proposition (blue = 10) from QBank to EBank. An ACCEPT of that proposition (e.g., *I agree*) then moves it from EBank to FBank, and removes inconsistent propositions (e.g., blue = 20, blue = 30, etc.) from the CGS.

**Figure 2:**
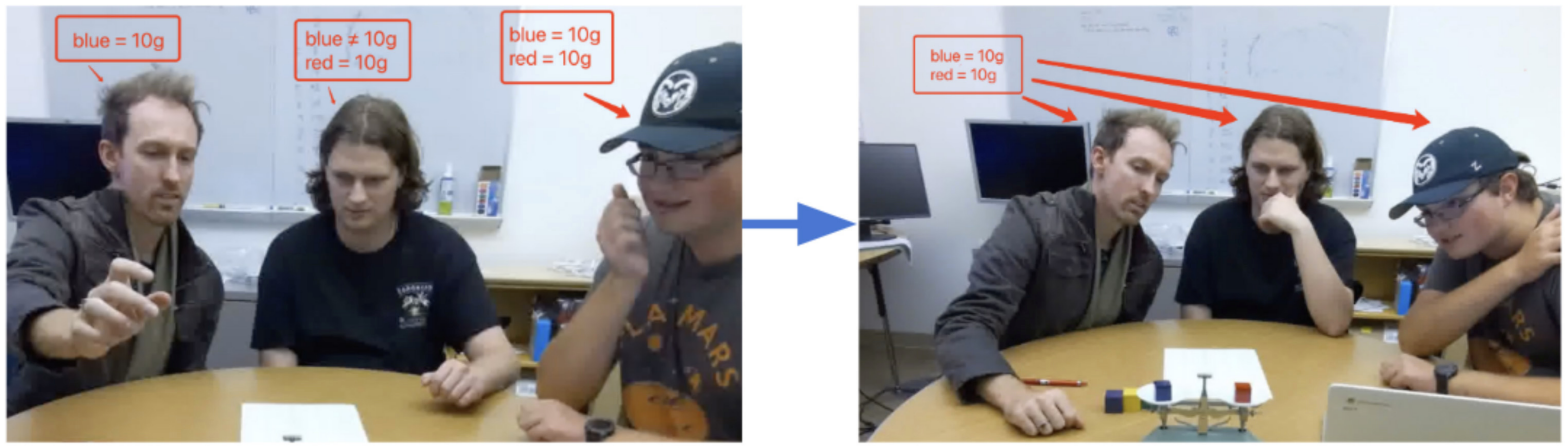
Example of a common ground update in the Weights Task. **(Left)** P1 believes “blue=10g”, but does not agree that “red=10g.” **(Right)** After seeing the scale, P1, P2, and P3 all agree on both propositions.

As an example, in Figure 2, the participants have a shared belief that the blue block weighs 10 grams, while it is not yet common knowledge that the red block weighs 10 grams. In other words, blue = 10 is in FBank, while red = 10 is in QBank. After putting the blue and red blocks on the scale and observing that the scale is balanced, participant 1 says “Yeah OK so now we know that this is also ten”. This moves red = 10 from QBank to EBank. Participant 2 then says “OK”; this promotes red = 10 from EBank to FBank.

## 6 Experiments

**Figure 3:**
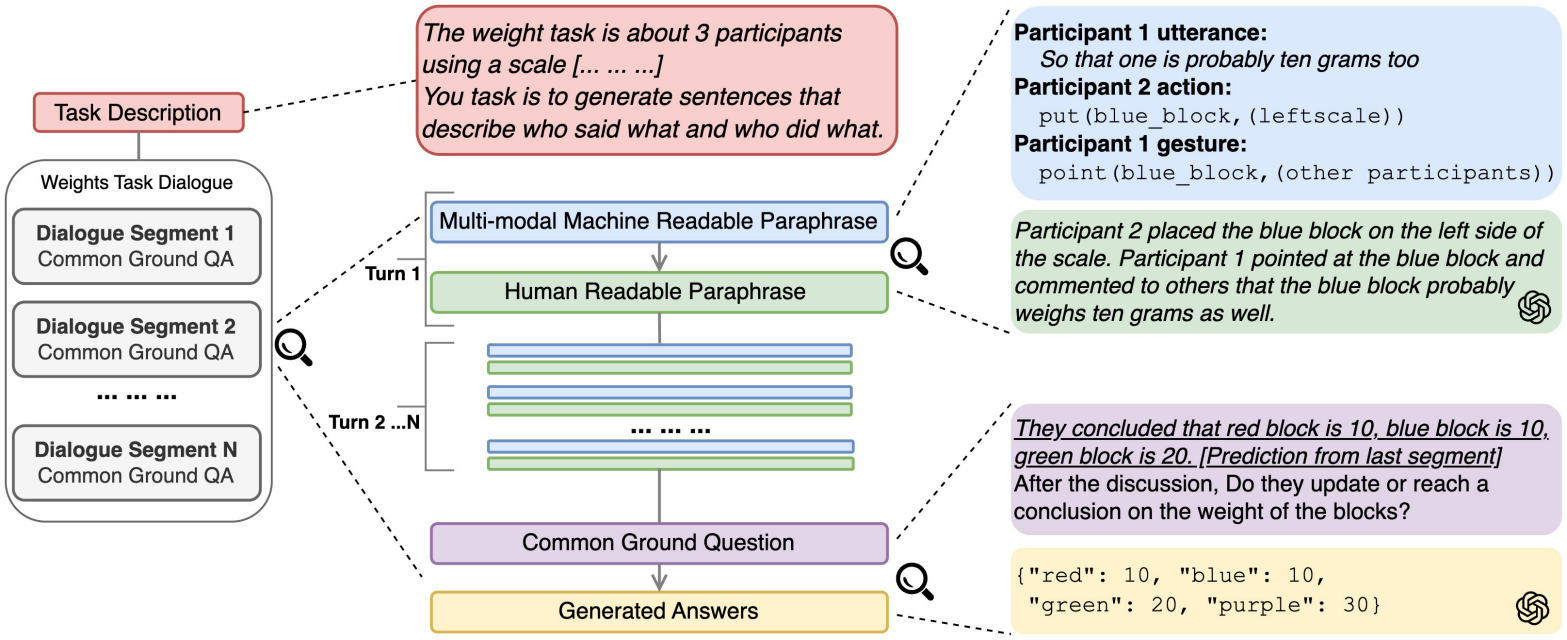
Common ground tracking pipeline with LLMs. Text format and emphasis on model input are addded for clarity.

In this section, we present experiments on the CGT task by applying our proposed MMDP pipeline (Section 6.2.4) on the Weights Task Dataset under a zero-shot learning setting. At a high level, we formalize CGT as a closed-domain QA task, where the language model is prompted with the evidential context from a dialogue segment and a question asking about the established common ground regarding the block weights. Based on the DP outputs, the context for each question also includes the natural language utterance paraphrases of all previous turns from the beginning of the dialogue. At each turn, the question includes the model prediction of the CG from the last dialogue segment (underscored text in Figure 3).^2^ We also instruct the model to generate the prediction in JSON format, so that it can be easily incorporated into the question prompt or processed for the evaluation. We experiment with GPT-3.5 (Brown et al., 2020) for both the DP and QA steps for its accessibility and cost-efficiency. We use the OpenAI API version gpt-3.5-turbo-0125. Finally, we use the Dice Similarity Coefficient (DSC) as the evaluation metric (Sørensen, 1948; Dice, 1945). DSC is similar to F1 score, measuring the similarity between gold and predicted common ground propositions.

### 6.1 Design

We propose a new method, MMDP, that can improve the CGT task by utilizing language only LLMs. Instead of using Multimodal LLMs that consist of different encoders to encode information from multiple modalities (Yin et al., 2023), we extend DP to the action and gesture annotations from the WTD. We leverage the capability of LLMs to paraphrase multimodal input into a natural language form, and infer the common ground from the dialogue context. Figure 3 illustrates our proposed LLM-prompting pipeline for modeling the CGT task. In the rest of this section, we first describe the data preprocessing pipeline (Section 6.2) where the DP techniques are used, and then the description of the prompt design and major components that use the paraphrases.

### 6.2 Data preprocessing pipeline

We describe the data selection and processing pipeline on the WTD to prepare conversational inputs to the model. The source of the annotations described in this section is a combination of Khebour et al. (2023) and Khebour et al. (2024). Using our preprocessing pipeline, we experiment with primarily two subtasks (anaphora resolution and multimodal alignment) of DP as the implementation of the proposed MMDP.

#### 6.2.1 Speech

The speech audio from the WTD is segmented into utterances delimited by silence. Each utterance is manually transcribed, and we refer to this set of text as “raw” utterances. In addition, to enhance the CGT performance of the LLM, we decontexualize pronouns of task-relevant entities in the dialogue through coreferential redescription. We believe this DP method of redescription can link the same entities across different modalities and serve as an alignment in our uni-modal system. Following our previous work (Rim et al., 2023; Tu et al., 2023), we paraphrase the mentions that refer to the same entity into their most informative form, i.e., proper nouns. In example 1, we paraphrase “that one” into “the blue block” for systems to better understand the context.

(1) **P1 utterance**: Maybe we would put that one there too.
**P1 utterance with DP**: Maybe we would put *the blue block* there too.

This enriched set of text is referred to as “decontextualized” utterances in the rest of the paper. In our experiment, we use both the raw and decontextualized utterances, to measure the impact of DP.

#### 6.2.2 Actions

The WTD provides manual annotations of agentive actions regarding block placement. The annotation is done in semi-logical, parenthesized form, but we found some annotation errors while experimenting. Hence we decided to review the entire action annotation, and manually fixed the found errors. Most of the errors we found were missing annotations when multiple blocks were moved together, but also a smaller number of duplicates and incorrect block color markings were found.

#### 6.2.3 Gesture

We convert the gesture annotation from GAMR syntax to “enclosed” text with parentheses to mark up patterns that can be more efficiently interpreted by language models (Zhai et al., 2022; Zhang et al., 2023). This also made the syntax more consistent with the VoxML-based action annotations when aligned together. We adopt a heuristic method to map the gesture acts from the datasets to their closest event head (e.g., deixis-GA to *point*, emblem-GA to *confirm*), and parse the gesture graph to extract the corresponding arguments. Specifically, for example in 2, we map the deictic act to the pointing action, and remove the argument name and variable to keep it simple in the input.

(2) **GAMR**:
    (d / deixis-GA
      :ARG0 (p1 / participant_1)
      :ARG1 (b / blue_block)
      :ARG2 (g / group))
    **Enclosed:**
    point(blue_block,(other participants))

#### 6.2.4 Multimodal alignment

Following the same setting in Khebour et al. (2024), we align the actions and gestures with the utterance that overlaps the most in terms of the starting and ending times. As briefly discussed in Section 3.2, we use two different forms of linguistic paraphrasing, the Machine Readable Paraphrase (MRP) and Human Readable Paraphrase (HRP), to obtain alignment of information across different modalities.

Specifically for this work, MRP is a form of (semi-)structured textual representation of the multimodality being expressed in the dialogue. Concretely, we generate an MRP of a multimodal dialogue segment as a set of key-value pairs that map each agent and modality to the content of the communicative event (e.g., action, utterance, gesture, etc.). While doing so, we apply some normalization to the raw annotation (Section 4). MRP features a uniform structure and text patterns that efficiently encode the semantics of the multimodal interactions in a dialogue. It also provides a pluggable expansibility for additional modalities, by adding or removing keyed pairs from the structure.

The second step of MMDP is the conversion from MRP to HRP with the application of LLMs. Compared to the MRP, the HRP in its natural language form is more effective to be processed and interpreted by language models. Similar to the paraphrases from DP, the HRP also encodes implicit semantics, enabled by LLMs' capabilities to reconstruct sentence structures of the (often incomplete and disfluent) speech and to resolve anaphoric references across different modalities. This can help generate more coherent paraphrases. We show how HRP conversion is done and then show the utility of MMDP by applying it on WTD in the following sections.

#### 6.2.5 Dialogue segmentation

In the CGT task, we focus on identifying the common ground that is updated right after the ACCEPT dialogue move. The ACCEPT move is essential in establishing the common ground in the whole dialogue, and previous work (Khebour et al., 2024) finds that it is more challenging to model the ACCEPT move than the other moves. We split the dialogues into segments on the ending time of each ACCEPT move. We show the number of ACCEPT moves (segments) and utterances in Table 1. On average, each group is annotated with 4.5 ACCEPTs. The group with the most ACCEPTs has six segments and the least, 2. The average number of utterances in each group is 43.4 where group 7 has the most utterances (54) and group 9 has the least (19).

**Table 1:** Statistics of accepted statements and utterances in CGA.

	**Count**
# of groups	10
Avg. # of utterance per group	43.4
Min / max # of utterances	19/54
Avg. # of ACCEPT moves per group	4.5
Min / max # of ACCEPT moves	2/6

### 6.3 Experiments with Large Language Models

#### 6.3.1 In-context task instructions

We apply the LLMs on the CGT task under an in-context learning scenario. We first manually generate the Weights Task description of the situated task setting (red unit in Figure 3), and use it as the system prompt input to the model. Within each segment of dialogue that establishes common ground, we create a prompt for each turn with the multimodal MRP that is converted from the existing annotations, and ask the model to generate an HRP in a natural language form. At the end of each dialogue segment, we instruct the model to infer the current common ground over the block weights by prompting it with the question.

#### 6.3.2 Dense paraphrasing of multimodal input

As shown in Figure 3 (blue unit), given the aligned annotations, we create an MRP as a key-value pair structure, where the key encodes the speaker ID and the modality, and the value encodes the annotation contents, normalized for non-speech modalities (Section 4). This set of pairs is then serialized into a concatenated string representation, which we call MRP.

(3) **P1 utterance**: Maybe we would put that one there too.
**P1 gesture**: point(blue_block,(other participants))

**Figure 4:**
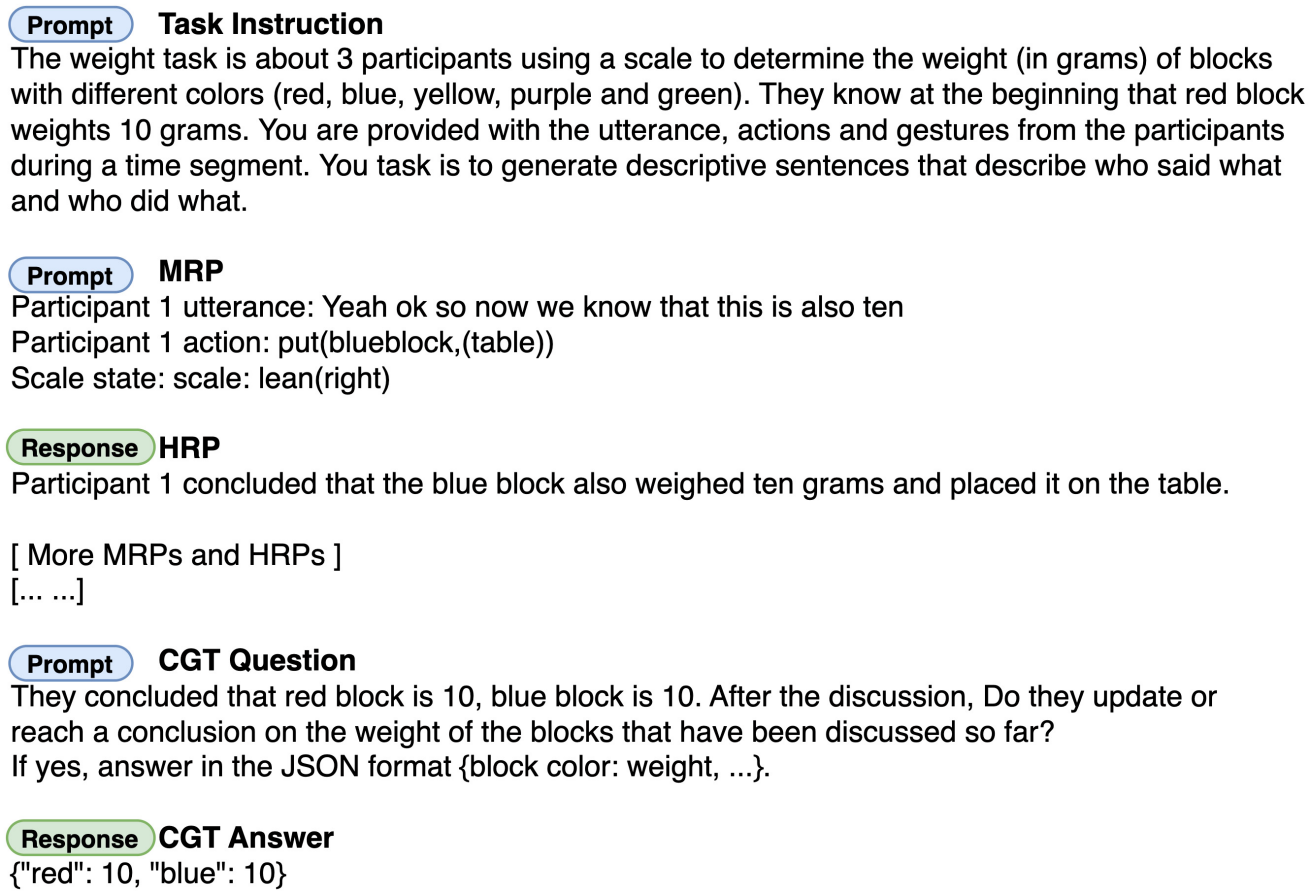
Full prompt to the LLMs for the common ground tracking pipeline. Text format and emphasis are added for clarity.

Example 3 shows a sample utterance with an aligned gesture, transformed to an MRP. After the MRP is constructed, we apply the language model to convert it to an HRP (Section 6.2.4). In order to generate the HRP from each turn, the current MRP along with all the HRPs from previous turns starting from the beginning of the dialogue are included in the context prompt. Figure 4 shows the full prompt for the CGT pipeline. The data input is changed accrodingly to accommodate different experiment settings.

#### 6.3.3 Baseline settings

We evaluate our approach against CGT baselines across three input settings: language-only, all-modalities in textual form, and all-modalities incorporating both text and images. For language-only and all-modalities in textual form, we employ baseline models from Khebour et al. (2024). In the language-only scenario, Khebour et al. (2024) transform decontextualized utterances (Decont.) into embeddings and utilize a similarity-based method to identify the common ground. For the all-modalities in textual form setting, a hybrid method is used which involves human annotations to map predicted utterance IDs to the corresponding common ground.

In addition to textual input, our method capitalizes on LLMs to reason with both text and images. Specifically, we extract five image frames evenly from each utterance's corresponding video clip and use these frames together with the utterances as input to incorporate multimodal information.^3^ For this setting, we apply GPT-4o and GPT-4o-mini as baseline models.

### 6.4 Results

Table 2 compares the CGT results between the baseline models and our methods under different settings. Under the language-only setting, DP-Utt. and DP-Decont. use raw and decontextualized utterances, respectively, in our pipeline without the paraphrasing step. Compared to the baseline results that use the decontextualized utterances as input, DP-Utt. is able to achieve comparable results (0.6 points lower) without access to the decontextualized information, suggesting LLMs are better at learning from the conversation context. However, by using the same decontextualized utterances as the input, DP-Decont. outperforms the baseline by a large margin (20.4 points).

**Table 2:** Evaluation results on the CGT task.

	**Group 1**	**Group 2**	**Group 3**	**Group 4**	**Group 5**	**Group 6**	**Group 7**	**Group 8**	**Group 9**	**Group 10**	**Avg**.
**Language-only**											
Baseline (Khebour et al., 2024)	0.0	52.8	**50.1**	4.5	16.5	**37.2**	**82.5**	52.6	0.0	0.0	29.6
DP-Utt.	74.8	39.9	37.5	0.0	56.1	6.1	0.0	47.4	0.0	28.6	29.0
DP-Decont.	**90.1**	**58.0**	35.8	**45.0**	**66.7**	17.2	61.8	**60.6**	**3.6**	**60.9**	**50.0**
**All-modalities in textual form**											
Baseline (Khebour et al., 2024)	42.5	48.0	**41.8**	34.8	31.8	**31.5**	**63.7**	57.4	0.0	**79.4**	43.1
MMDP-Utt.	85.0	36.5	37.5	38.2	54.3	0.0	55.2	48.7	0.0	63.7	41.9
MMDP-Decont.	**88.3**	**58.0**	35.8	**45.0**	**65.2**	17.2	55.2	**63.3**	**13.8**	71.6	**51.3**
**All-modalities in text and video frames**											
Baseline-GPT-4o-mini	55.3	33.1	0.0	32.1	38.0	26.5	0.0	48.7	0.0	31.0	26.5
Baseline-GPT-4o	84.1	33.1	34.0	32.1	47.8	26.5	0.0	43.3	0.0	43.8	34.5

DSC is reported for each group and the average under multimodal and language only settings. The bold value indicates the best DSC under different settings.

Under the setting of all-modalities in textual form, the Baseline adopts a hybrid method that uses annotations to map predicted utterance IDs to the corresponding common ground. MMDP-Utt. combines the action, gesture and raw utterance in the MRP as the input. Similarly, MMDP-Decont. uses the decontextualized utterance in the MRP instead. Compared to DP-Utt., MMDP-Utt. improves the results by 13 points, suggesting the usefulness of multimodal information for the CGT task. Both DP-Decont. (6.9 points) and MMDP-Decont. (8.2 points) perform better than the stronger multimodal baseline. Compared to DP-Decont., MMDP-Decont. performs only slightly better by incorporating additional annotations from other modalities (1.3 points). This may suggest that the decontextualized utterances have already encoded most of the multimodal information, and MMDP-Decont. exhibits an upper-bound performance for the CGT task.

Compared to representing multimodalties in MRP, using video frames as additional input does not exhibit better performance over our proposed method. Under this setting, baseline with GPT-4o outperforms GPT-4o-mini, yet it is still worse than MMDP-Utt. which integrates action, gesture and raw utterance in the MRP (7.4 points lower). Overall, we show the effectiveness of our LLM pipeline, and the decontextualized utterances enhanced with multimodal textual paraphrases can yield the best results for the task.

#### 6.4.1 Error analysis

While the dialogues are all about the Weights Task in the dataset, the conversations from different groups exhibit various patterns that are also reflected in the CGT results. We briefly characterize the cases where the performance from the baselines and our methods have salient gaps on individual groups.

The MMDP method improves the most on group 1 (90.1 points for language only, 45.8 points for all modalities). By examining the dialogue, we find that this group builds up the common ground in a “bottom-up” style by identifying the block weights from the lightest to the heaviest. This way the conversation depends heavily on the context, making MMDP a better choice to capture these long dependencies. In addition, all modalities in this group play important roles in identifying the common ground.

(4) **P2 utterance**: That's ten so then
**P1 action**: put(blue_block,(left_scale))
**Common ground**: blue = 10

(5) **P2 utterance**: Probably thirty at this point
**P1 action**: point(purple_block,(other participants))
**Common ground**: purple = 30

Consider example 4. The utterance from Participant 2 mentions the possible weight of a block, and the aligned putting action from Participant 1 indicates that the block is blue. Similarly in example 5, The pointing gesture also indicates the weight from the utterance is for the purple block.

Although our method improves the overall performance, the baseline performs better on group 6 (20 points for language only, 14.3 points for all modalities). Unlike group 1, we observe that the dialogue from this group contains many implicit assumptions that are not expressed either verbally or non-verbally. This makes the annotation quite sparse and difficult for LLMs to build up the conclusion from the context. This pattern also appears in group 3. Participants also sometimes refer to the color of the block in a non-standardized way, which causes further confusion for the model.

(6) **P2 utterance**: So big blue is probably thirty
**Common ground**: purple = 30

In example 6, participant 2 refers to the color of the purple block as “big blue” throughout the whole dialogue.

CGT on the dialogue from group 9 is challenging to both the baseline and MMDP. After examining the data, we notice that most action and gesture annotations are not aligned with the utterances, making the improvement from multimodal information incremental. This may be due to the nature of the conversation where non-verbal actions happen asynchronously with the utterance. In addition, the less frequent usage of pronoun references in this dialogue makes it difficult to take advantage of the decontexualization of the utterances.

(7) **P3 utterance**: Looks equal yeah
**P2 utterance**: Yeah that's good
**P1 utterance**: Look we have the thirty gram block

Example 7 shows the key utterances for establishing the common ground from group 9. The lack of proper multimodal alignments and block references poses a lot of challenges to the CGT automation.

Multuimodal GPT with both text and image input performs worse than textual MRP and HRP. This could be attributed to the insufficient salient mappings between videos frames and the corresponding utterance. Notably in Group 7, where the models struggle to identify the correct common grounds, many actions (e.g., *slightly lift the block and then put it back on the scale*) involve quick and subtle movements that are challenging for the models to accurately capture. Moreover, gestures in the video can be inherently ambiguous, especially when a participant points to a specific block that is positioned near other blocks. However, the converted MRP from the multimodal input is useful in providing accurate information and eliminating the ambiguities from the video frames.

## 7 Discussion and analysis of MMDP

In this section, we further explore the utility of the MMDP method. We experiment with MMDP on the CGT task, and conduct quantitative analysis of the results with different model selection and input data variance.

### 7.1 Larger language models

We evaluate a larger and more powerful language model in the MMDP pipeline. We apply GPT-4o (OpenAI, 2023) for both the DP and QA steps. We use the OpenAI API with version gpt-4o-2024-05-13. Table 3 shows the model comparison results. Overall, GPT-4o performs better than GPT-3.5 when decontextualized or multimodal information is provided in the input. However, GPT-4o does not show superior results on the DP-Utt. setting. This confirms our findings that the richness of the multimodal information is essential to resolve the CGT task.

**Table 3:** Evaluation results on the CGT task.

	**DP-Utt**.	**DP-Decont**.	**MMDP-Utt**.	**MMDP-Decont**.
GPT-3.5	**29.0**	50.0	41.9	51.3
GPT-4o	28.6	**53.8**	**45.8**	**54.9**

We compare GPT-3.5 with GPT-4o under different pipeline settings. Average DSC over all groups is reported. The bold value indicates the best DSC under different settings.

### 7.2 Multimodal information encoded with HRP

In the MMDP pipeline, we propose a DP step that converts the multimodal MRP into HRP. We explore the utility of the DP step by using MRP vs. HRP as the model input. Table 4 shows the evaluation results. In general, models with HRP perform better than those with MRP, suggesting the effectiveness of DP in grounding non-verbal information into language form. Compared to GPT-3.5, applying DP with GPT-4o results in less differentiation in the performance (3.1 vs. 7.5). This indicates that a larger language model has more capabilities to learn structured information from MRP directly.

**Table 4:** Evaluation results from the GPT models under the multimodal setting.

**Setting**	**Model**	**Use HRP**	**DSC**
MMDP-Utt.	GPT-3.5	✘	34.4
	GPT-3.5	✔	41.9
	GPT-4o	✘	42.7
	GPT-4o	✔	45.8
Baseline^‡^	N/A	N/A	43.1
MMDP-Decont.	GPT-3.5	✘	47.3
	GPT-3.5	✔	51.3
	GPT-4o	✘	52.9
	GPT-4o	✔	54.9

We compare the DSC with or without the DP step for HRP generation. ^‡^Baseline from all modalities.

### 7.3 Dialogue context cutoff

We evaluate whether MMDP can enable more efficient learning by cutting off the previous dialogue context in the input. In our current pipeline, in the prompt for every DP and QA step, we include previous generated HRPs and common ground predictions from the *beginning* of the dialogue. In this experiment, we only keep the HRPs from the *current* dialogue segment in the prompt. Table 5 shows the evaluation results. In general, we notice a performance drop under most settings after applying the context cutoff. Although the question prompt still has access to the previous common ground prediction, the limited context poses additional challenges to the model. MMDP-Decont. has the highest drop (6.2) in performance. This may be because the combination of decontextualized utterance and multimodal information from the bigger context contributes the most to model performance. DP-Utt. shows a similar result with the cutoff. This may result from the already existing lack of annotation in the context of raw utterances. Overall, we observe that although there exists a trade-off between performance and efficiency, the model with context cutoff is still able to produce competitive results compared to the baseline (43.1).

**Table 5:** Evaluation results on the CGT task.

	**DP-Utt**.	**DP-Decont**.	**MMDP-Utt**.	**MMDP-Decont**.
Cutoff	28.7	50.3	43.9	48.7
No-cutoff	28.6	53.8	45.8	54.9

We apply GPT-4o and compare the average DSC with or without context cutoff.

### 7.4 Re-annotation of CGA

Since the size of the CGA is limited, we provide additional annotations for future research. Specifically, in our experiments, we find that STATEMENTs are often not followed by explicit ACCEPTs. This results in propositions remaining in EBank and not moving to FBank, even when the dialogue continues as if the participants all believe the stated proposition. For this reason, we add an implicit ACCEPT to each STATEMENT in the CGA, except those that are followed by a DOUBT. This can be seen as allowing most STATEMENTs to directly promote propositions from QBank to FBank. The re-annotation increases the average number of ACCEPTs from 4 to 14. The smallest increase is from 2 to 7 ACCEPTs. The most significant increase is observed in Group 5 that raises the number of ACCEPTs from 3 to 17. Table 6 shows the number of ACCEPTs in the original and re-annotation of CGA.

**Table 6:** Number of ACCEPTs in the original and re-annotation of CGA.

	**Original**	**Re-annotation**
Group 1	6	15
Group 2	5	16
Group 3	4	16
Group 4	2	7
Group 5	5	18
Group 6	3	17
Group 7	4	10
Group 8	6	16
Group 9	4	11
Group 10	6	20
All	45	146

We run the same experiments on the new CGA data using GPT-3.5. Table 7 shows the results. Although not directly comparable because of the different number of ACCEPTs, we notice that the average DSC on the re-annotated data is over 20 points higher than that on the original dataset. The results improve the most under the DP-Decont. setting (32.2 points higher). Overall, we find that using a less strict rule to identify ACCEPTs, and as a result, more accepted statements can lead to significant improvements on the CGT task. We suspect that the improvements stem from more ACCEPTs that agree with the same STATEMENT being annotated; e.g., there is only one ACCEPT of STATEMENT red = 10 in the original data. In the new data, two more ACCEPTs of the STATEMENT are annotated without any additional ACCEPTs to the other STATEMENTs.

**Table 7:** Evaluation results from GPT-3.5 on the CGT task with re-annotated CGA.

	**DP-Utt**.	**DP-Decont**.	**MMDP-Utt**.	**MMDP-Decont**.
Original	29.0	50.0	41.9	51.3
Re-annotation	56.4	82.1	67.3	75.5

Average DSC over all the groups are reported.

### 7.5 Limitations

One limitation of our work comes from the dataset selection, as our study of the CGT is solely based on the Weights Task Dataset (WTD). WTD contains ten recorded dialogues in a controlled setting, where three participants collaborate on a weight task to reach common ground. While the WTD provides a detailed view for examining human interactions over multiple communication modes, it may not fully capture the diversity found in real-world situations. Due to the small size of the dataset and the controlled task setting, the effectiveness of our MMDP method in understanding and tracking common ground may not easily extend to interactions that differ significantly from those in the WTD. To our best knowledge, WTD is the only exisiting CGT dataset. Future work could focus on expanding the dataset size and incorporating more diverse dialogues within other problem-solving task settings, such as tangram puzzles. Our experiments on the WTD involve dialogues in English only. Future studies involve exploring CGT in multilingual contexts.

## 8 Conclusion

In this work, we have highlighted the importance of integrating multimodal representations in the development of more sophisticated and accurate dialogue systems, particularly in the service of addressing underspecified references within cross-modal settings. We proposed MMDP by extending the technique of DP for converting the annotations from multiple modalities into textual paraphrases with both machine-readable and human-readable formats. We built an LLM-based pipeline by applying MMDP on WTD, and showed that the generated paraphrases can be used effectively to improve performance on the CGT task under different model settings. We conducted a quantitative analysis of the results from experiments with different models, paraphrase input and context length, and showed that MMDP could still show competitive performance even with limited information from the input. We believe that MMDP for enhancing the interpretative power of multimodal dialogue systems constitutes a step toward a more capable and competent human-computer interaction in multimodal environments.

## Data Availability

The raw data supporting the conclusions of this article will be made available by the authors, without undue reservation.

## Footnotes

^1^ https://github.com/brandeis-llc/mmdp-cgt.git

^2^An exception is the question for the first dialogue segment, for which there is no previous prediction.

^3^The average video clip length corresponding to each utterance is 4.3 s, with the longest being 21, 18, and 13 s, respectively. We believe that using five frames per utterance effectively captures the action and event dynamics occurring within the duration of each utterance.
